# Evaluation of the Persistence and Characterization of *Listeria monocytogenes* in Foodservice Operations

**DOI:** 10.3390/foods11060886

**Published:** 2022-03-20

**Authors:** Magaly Toro, Jessica Williams-Vergara, Camila Solar, Ana María Quesille-Villalobos, Hee Jin Kwon, Paola Navarrete, Jianghong Meng, Yi Chen, Angélica Reyes-Jara

**Affiliations:** 1 Laboratorio de Microbiología y Probióticos, Instituto de Nutrición y Tecnología de los Alimentos (INTA), Universidad de Chile, Avenida El Líbano 5524, Macul, Santiago 7810000, Chile; magaly.toro@inta.uchile.cl (M.T.); jwilliamsvergara@trentu.ca (J.W.-V.); camila.solar@inta.uchile.cl (C.S.); aquesille@inta.uchile.cl (A.M.Q.-V.); pnavarre@inta.uchile.cl (P.N.); 2 Department of Nutrition and Food Science, University of Maryland, College Park, MD 20742, USA; hkwon22@umd.edu (H.J.K.); jmeng@umd.edu (J.M.); 3 Joint Institute for Food Safety and Applied Nutrition (JIFSAN), University of Maryland, College Park, MD 20742, USA; 4 Center for Food Safety and Applied Nutrition, Food and Drug Administration, College Park, MD 20740, USA; yi.chen@fda.hhs.gov

**Keywords:** *Listeria monocytogenes*, foodservice operations, GMP, biofilm, quaternary ammonium resistance, genomic analysis

## Abstract

*Listeria monocytogenes* is a major foodborne pathogen that can contaminate food products and colonize food-producing facilities. Foodservice operations (FSOp) are frequently responsible for foodborne outbreaks due to food safety practices failures. We investigated the presence of and characterized *L. monocytogenes* from two FSOp (cafeterias) distributing ready-to-eat meals and verified FSOp’s compliance with good manufacturing practices (GMP). Two facilities (FSOp-A and FSOp-B) were visited three times each over 5 months. We sampled foods, ingredients, and surfaces for microbiological analysis, and *L. monocytogenes* isolates were characterized by phylogenetic analyses and phenotypic characteristics. GMP audits were performed in the first and third visits. A ready-to-eat salad (FSOp-A) and a frozen ingredient (FSOp-B) were contaminated with *L. monocytogenes,* which was also detected on Zone 3 surfaces (floor, drains, and a boot cover). The phylogenetic analysis demonstrated that FSOp-B had persistent *L. monocytogenes* strains, but environmental isolates were not closely related to food or ingredient isolates. GMP audits showed that both operations worked under “fair” conditions, and “facilities and equipment” was the section with the least compliances. The presence of *L. monocytogenes* in the environment and GMP failures could promote food contamination with this pathogen, presenting a risk to consumers.

## 1. Introduction

*Listeria monocytogenes* is a foodborne pathogen responsible for causing listeriosis, a low-incidence disease with a high mortality rate (20–30%) [1,2]. The invasive form of listeriosis affects vulnerable populations such as immunocompromised individuals, pregnant women, infants, and the elderly. The pathogen can cause febrile gastroenteritis in healthy individuals if consumed in large amounts [3,4]. Consequently, listeriosis is considered a serious public health concern.

This bacterium can survive and grow in harsh environmental conditions such as those used in food processing plants. *L. monocytogenes* can persist for months or years in food processing environments; it can colonize niches within facilities such as cracked surfaces, drains, and areas that are hard to clean [5,6,7]. Consequently, *L. monocytogenes* have been isolated from floors, wastewater pipes, improperly cleaned and sanitized equipment, and even cooked foods [7,8]. This widespread distribution is directly related to *L. monocytogenes*’ ability to form biofilms since these structures protect microorganisms embedded in its polymeric matrix. Cells within biofilms are more resistant to stress conditions such as cleaning and disinfection [9,10,11]. Studies show that *L. monocytogenes* exposure to sub-inhibitory concentrations of disinfectants based on quaternary ammonium compounds (QACs) can increase the speed of biofilm formation [10,12]. Some genes linked to biofilm formation overexpress in QAC-resistant *L. monocytogenes* [13,14,15].

Persistent contamination of *L. monocytogenes* in food process environments plays an essential role in the contamination of processed food products [16,17,18]. The presence of persistent strains can be identified by isolating highly genetically related isolates in the same plant at intervals of 6 or more months [19]. Previous reports have used serotyping and ribotyping to define the presence of such isolates [20,21,22,23]. The current use of whole-genome sequencing and bioinformatics analysis provides an excellent tool to subtype bacteria with high-resolution power and establish phylogenetic relationships between isolates better than any other method. Genomic analysis also delivers valuable data to characterize isolates, such as identifying virulence genes, serotypes, sequence types, and antimicrobial resistance genes [24,25,26,27,28].

Contamination of food products with *L. monocytogenes* can occur at any processing stage, including food service operations (FSOp), where foods are handled, prepared, and directly served to customers [6]. Thus, food service environments can become a source of contamination with *L. monocytogenes,* potentially a risk to human health. Therefore, FSOp must implement food safety procedures such as good hygiene practices and sanitation programs to guarantee safe foods, among other considerations [29]. Control strategies and adequate sanitization procedures are essential in processing environments to prevent food contamination with foodborne pathogens such as *L. monocytogenes*. The most effective methods to reduce *L. monocytogenes* contamination are implementing good manufacturing practices (GMP) to prevent cross-contamination and carrying out intensive environmental sampling programs [16,30]. Plans implementing monitoring audits allow assessing critical stages in food production and evaluating risk factors for the contamination of foods; therefore, they provide a valuable tool to prevent food contamination [31,32].

In this study, we evaluated the presence of *L. monocytogenes* in two FSOp (cafeterias) in the Metropolitan Region of Santiago, Chile. This study aimed to evaluate the contamination of *L. monocytogenes* in two FSOp across time and identify persistent strains in their environment. We also assessed the compliance of these FSOp with GMP to identify risk factors that would favor *L. monocytogenes* contamination.

*L. monocytogenes* was detected in food ingredients, ready-to-eat salad, and the environment of two foodservice operations (cafeterias). The genomic analysis demonstrated that some persistent *L. monocytogenes* strains were present in drains and spread to surfaces closer to foods. Finally, multiple non-compliance issues with GMP were found in both operations, especially related to facilities and equipment, which might impact their final products’ microbiological quality.

## 2. Materials and Methods

### 2.1. Description of Food Services Establishments

**Figure 1:**
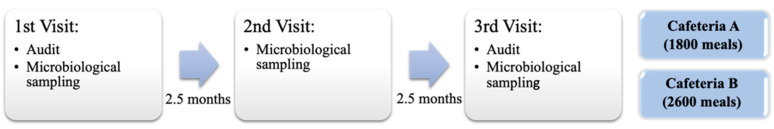
Visits scheme to FSOp-A and FSOp-B.

We monitored two privately owned FSOp: a medium (A) and a large (B) cafeteria. Approximately 1800 meals were prepared each day in FSOp-A and 2400 in FSOp-B (Figure 1). Meals were prepared and cooked on-site and served directly to the users at their dining hall/cafeteria. Both FSOp included two production lines: a hot kitchen (for cooked food preparation) and a cold kitchen (for food served cold). The study was carried out over five months, and each operation was visited two times for audits and three times for microbiological samplings. Details of these visits are presented in Figure 1.

### 2.2. Microbiological Sampling

#### 2.2.1. Food Products Sampling

A total of 72 food samples were obtained from the two facilities (FSOp-A = 9 and FSOp-B = 15 in each sampling visit). Samples considered both ingredients and prepared meals. Ingredients were selected focusing on ready-to-eat (RTE) goods (e.g., packaged cut celery, cold cuts, frozen avocado). Meals were selected based on their availability for the users at the visits, which were carried out at lunch—the main mealtime in Chile. Meals served hot (above 68 ℃) right after preparation were not sampled (such as stews). Meals served cold, and those that did not require re-heating before serving were selected as prepared meal samples (e.g., beef and mixed vegetable salad, egg and lettuce salad, among others). Desserts were classified as cold dishes (e.g., Spanish custard, rice pudding, fruit salad) (Table S1).

#### 2.2.2. Environmental Sampling

A total of 146 surface samples were taken in the facilities. We obtained 20 surfaces samples at each visit of FSOp-A and 26 (first visit) and 30 (visits 2 and 3) surface samples at FSOp-B (Table 1). Surface samples were obtained in different facility areas according to environmental zoning and considering areas with the highest to the lowest risk of food contamination: Zone 1 surfaces that were in direct contact with food products (e.g., knives, cutting boards, counters.); Zone 2 areas adjacent to Zone 1 (e.g., refrigerators, walls, aprons); Zone 3 areas surrounding Zone 2 inside the production area (e.g., drains, door handles, ceiling); Zone 4 areas outside of food processing zones (e.g., dressing rooms, bathrooms, offices) [33]. Zone 4 samples were eliminated in visits 2 and 3 due to results indicating the absence of *L. monocytogenes* and the low risk they represented (Table 1). For flat food-contact surfaces (e.g., floor, worktable), a template was used to define an area of 10 cm^2^, which was sampled by swabbing vertically, horizontally, and diagonally ten times in each direction. Irregular shape surfaces (e.g., drains, door handles, trolley wheels) were swabbed at least ten times, moving up and down, covering the entire surface. Swabs were transported in 10 mL of Letheen neutralizing broth below 8 °C and taken to the laboratory in less than 4 h, where samples were analyzed within 12 h of arrival.

### 2.3. Microbiological Analysis and Bacterial Identification

#### 2.3.1. Microbiological Analysis of Food Samples

Microbiological tests for food samples were performed in conformity with the requirements of the Chilean Food Safety Regulation (*Reglamento Sanitario de los Alimentos*; RSA) (e.g., pre-processed vegetables, RTE cold served meals) [34]. Briefly, 25 g from each sample were aseptically transferred into a sterile plastic bag and homogenized in a food mixer (Bag Mixer 400, Interscience International, Saint-Nom-la-Bretèche, France) with 225 mL of sterile peptone water 0.1% (*w/v*) at 230 rpm for 2 min. Homogenized samples were serially diluted 10-fold with 9 mL of 0.85 % sterile saline. Dilutions were plated in different media according to the microbiological analysis to perform. For aerobic plate count, dilutions were plated onto Plate Count Agar (PCA) (Oxoid, CM0463 Hampshire, UK) and incubated at 30 °C for 48 h [35]. Enterobacteriaceae were determined using Violet Red Bile Glucose Agar (BD Difco™ 218661 Sparks, MD, USA), and plates were incubated at 36 °C for 18–24 h [36]. For *E. coli* enumeration, the most probable number protocol was used; samples were inoculated in lauryl sulfate broth (Oxoid, CM0451 Hampshire, UK). Tubes showing gas production and turbidity were positive for *E. coli* [37]. Values were calculated using the software https://mpncalc.galaxytrakr.org/, (accessed on 6 May 2021) proposed by BAM-FDA.

*S. aureus* enumeration was performed using Baird Parker agar with egg yolk tellurite emulsion (Oxoid, CM0275 Hampshire, UK) and incubated at 37 °C for 24 h [38]. *Clostridium perfringens* enumerations were performed using the pour plating method on Tryptose Sulfite Cycloserine agar (Oxoid, CM0543 Hampshire, UK) incubated in anaerobiosis at 36 °C for18–24 h [39]. *Bacillus cereus* was determined using the most probable number by the ISO21871 method [40]. Yeast and mold were enumerated onto Sabouraud Dextrose agar (BD Difco^TM^ 210950) incubated at 37 °C for 96 h according to ISO 21527–1:2008 (ISO, 2008) [41]. For *Salmonella* spp. detection, the ISO 6579-1 method was used [42], and colonies were verified by polymerase chain reaction (PCR) [43].

All food samples were analyzed for *L. monocytogenes* following the BAM protocol with minor modifications [44]. Samples (25 g) were homogenized and enriched in buffered *Listeria* enrichment broth (BLEB) (Oxoid, CM0897 Hampshire, UK) and incubated at 30 °C. After 24 and 48 h, BLEB enrichments were plated onto PALCAM (Oxoid, CM0877 Hampshire, UK) agar and Listeria Ottaviani and Agosti (ALOA) agar (Oxoid, CM1084, SR0226; SR0244; SR0228 Hampshire, UK) and incubated for 24 and 48 h at 37 °C. Presumptive *L. monocytogenes* colonies from each plate were confirmed by PCR using the following primers: lmo3F (5′-GTCTTGCGCGTTAATCATTT-3′) and lmo4R (5′-ATTTGCTAAAGCGGGAATCT-3′) [45]. For DNA extraction, 2-3 *L. monocytogenes* presumptive colonies were suspended in TE buffer (Tris-HCl 10mM; EDTA (Merck, Hamburg, Germany) 1 mM, pH 8), boiled for 10 min, and centrifuged at 10,000 rpm for 5 min; 2 μL of supernatant was used as a template for PCR reaction. PCR reactions contained 10 µL GoTaq^®^ Green Mastermix 2× (Promega, Madison, WI, USA), 0.5 μL (final concentration 0.3 mM) of each primer, and 7 μL molecular grade water to complete 20 μL final reaction volume. The PCR program included an initial denaturation at 94 °C for 5 min, 30 cycles of denaturation (94 °C for 30 s), annealing (58 °C for 30 s), and extension (72 °C for 30 s), and a final extension step at 72 °C for 5 min. *L. monocytogenes* EGD-e was used as a positive control. Confirmed isolates were stored in glycerol at −80 °C for further analysis.

#### 2.3.2. Microbiological Analysis of Environmental Samples

Environmental samples were analyzed for APC: one mL for each sample was serially diluted 10-fold with 9 mL of 0.85 % sterile saline. Results were expressed as CFU/cm^2^ or as CFU/total surface area for utensils and some equipment. Values described by Losito et al. [46] were used to define the acceptable level of microorganisms on clean food surfaces: APC bacterial counts for samples carrying levels ≤4.9 × 10^1^ CFU/cm^2^ were defined as “compliant”, samples between 5.0 × 10^1^ and 4.9 × 10^2^ CFU/cm^2^ were classified as “improvable”, and ≥5.0 × 10^2^ CFU/cm^2^ were “non-compliant”.

Environmental samples were analyzed for *L. monocytogenes*. For that, 5 mL of the sample (suspended in Letheen media) were enriched in 45 mL of BLEB and incubated at 30 °C. This culture was processed after 24 and 48 h of incubation, and procedures described in the previous section were followed for Listeria isolation.

### 2.4. L. monocytogenes Isolate Characterization

Isolates obtained from food samples and the environment were characterized as follows:

#### 2.4.1. Whole Genome Sequencing

Genomic DNA was extracted from an overnight culture in tryptic soy broth (TSB, BD Difco™ Sparks, MD, USA) with 0.6 % yeast extract (BD Difco™ Sparks, MD, USA) (TSBYE) media and extracted with the DNA blood and tissue kit using QIAcube (Qiagen Inc., Germantown, MD, USA), including a lysozyme digestion step. Libraries were prepared with the Nextera XT Library Preparation Kit (Illumina Inc., San Diego, CA, USA), and paired-end sequencing (2 × 300 bp) was performed in a MiSeq instrument using the Illumina MiSeq Reagent Kit v3 (600 cycles) following the manufacturer’s instructions. Sequence Read Archive data were uploaded to the National Center for Biotechnology Information.

#### 2.4.2. Genome Assembly and Genomic Characterization

Sequence reads were trimmed using Trimmomatic v0.36.4 [47] with default parameters. De novo assembly was performed using SPAdes v3.12.0 [48], and a qualitative evaluation of the assembly was performed by QUAST v5.0.2 [49]. Assemblies with good quality had a total length of 3.0 ± 0.3 Mb, N50 greater than 20 Kb, and contigs less than 500. In silico serogroups, sequence types (ST) and clonal complexes (CC) of *L. monocytogenes* were determined from their whole genome sequencing according to the Institut Pasteur *Listeria* database (https://bigsdb.pasteur.fr/listeria/, accessed on 4 Jun 2021). *L. monocytogenes* genomes were also analyzed for the absence/presence of genes encoding QAC resistance (*bcrA*, *bcrB*, *bcrC*, *qacC*, and *qacH*) and biofilm formation (*lmo2026*, *lmo0435 (bapL)*, *lmo0673*, *lmo2504*, *luxS*, and *recO*) [50] with Geneious Prime v2020.2.3 (https://www.geneious.com, accessed on 26 August 2021).

#### 2.4.3. Phylogenetic Relatedness among *L. monocytogenes* Isolates

Phylogenetic analyses were performed to determine the relatedness among *L. monocytogenes*. Analyses were run for all isolates and isolates belonging to the same serogroup obtained in each FSO. Pairwise SNP calling, filtering, and validation were performed with CSI Phylogeny 1.4 server from the Center for Genomic Epidemiology at Denmark Technical University (CGE: https://cge.cbs.dtu.dk/services/CSIPhylogeny/, accessed on 23 August 2021) with default parameters [51]. Neighbor-joining trees with 1000 replicates for bootstrap were constructed using a matrix of pairwise distances between strains using MEGA version X [52]. The reference genomes used for SNP analysis were as follows: *L. monocytogenes* EGD-e (NC_003210) was used for the analysis of all genomes; *L. monocytogenes* J1-220 (CP006046.4) was used for serogroup IVb, and strain J2 064 genome (CP006592.1) was used for serogroup IIb [53].

#### 2.4.4. Determination of the Minimal Inhibitory Concentration for Quaternary Ammonium (MIC-QA)

MIC-QA for *L. monocytogenes* isolates was determined through the agar dilution method and with a commercial sanitizer carrying 10% (100,000 ppm) quaternary ammonium (QA; Singen SQ-10^®^, Veterquímica, Chile). The recommended concentration for Singen SQ-10^®^ ranged from 200 to 500 ppm QA. Briefly, TSAYE plates were supplemented with seven 2-fold dilutions of QA, and final concentrations ranged from 2.5 ppm to 160 ppm QA. *L. monocytogenes* isolates were first grown in TSAYE plates overnight at 37 °C, and bacteria were suspended in a NaCl 0.9 % solution to an adjusted concentration of 10^6^ CFU/mL. Then, five μL of each culture were plated on the surface of the supplemented agar. The MIC-QA for each isolate was defined as the lowest QA concentration at which no growth was observed after overnight incubation at 37 °C.

#### 2.4.5. Evaluation of Biofilm Formation

The biofilm formation ability of *L. monocytogenes* isolates was evaluated using polystyrene plates as described by Ortiz et al. [11]. Briefly, *L. monocytogenes* strains were grown in TSBYE + NaCl (2%) + glucose (1%) at 37 °C. Cultures were adjusted to 10^8^ CFU/mL, and 200 μL were plated in quadruplicate into 96-well flat-bottom microplates. Microplates were incubated at 37 °C for 24 h; then, plates were washed three times with phosphate-buffered saline (PBS, pH 7.4), dried at room temperature, and stained with 0.5 % crystal violet for 15 min. After three washes with distilled water, 200 μL of 95 % ethanol was added to the plates, then read at 595 nm in an ELISA reader. Uninoculated TSBYE media was used as a blank.

### 2.5. Audits and Structure of the Checklist

Audits evaluated the compliance with prerequisites established by the Chilean RSA and GMP outlined in the national standard for FSOp [34]. Two of our GMP and Food Safety trained inspectors designed the checklist and applied it in situ. Data were collected using a structured checklist of 276 closed-ended questions. Possible answers for each question were “compliant, (score = 1)”, “non-compliant (score = 0)”, “partially compliant (score = 0.5)”, and “not applicable (the question was removed for compliance level calculation)”. The items evaluated included the following (n = question number and max score): facilities and equipment (n = 32), ingredient reception and storage (n = 33), processing practices (n = 120), personnel (n = 18), cleaning and sanitation (n = 14), waste management (n = 11), and documentation and records (n = 48) (Table S2). The information was collected by direct observation, interview of food handlers, and document verification with the on-site manager. Inspected areas included cold and hot kitchens, pre-processing areas for vegetables and meats, bakeries, storage areas, refrigeration chambers, and dining halls. Overall and item compliance levels were defined by the percentage of each section’s total score. Compliance levels were defined as “suitable” (90–100%), “fair” (76–89%), “regular” (66–75%), and “critical” (<65%).

### 2.6. Statistical Analysis

We used a logit model described by Hammons et al. (2015) [54] to determine the probability of detecting *L. monocytogenes* on surfaces based on its correlation with aerobic plate counts (APC). The APC level was expressed on a logarithm scale (Log APC). Equation (1) shows the model:

logit(*p*(y = 1)) = β0 + β1 × Log(APC) (1)

Aiming a straightforward interpretation of the model, we report the exp(β1), which indicates the changes in the odds ratios of finding *L. monocytogenes* associated with a one-unit increase in Log (APC). “y” is a binary variable, “y” = 1 denotes the presence of *L. monocytogenes,* and “y” = 0 absence of *L. monocytogenes*. We used a z-score to assess the significance of the coefficients (model coefficient, *p*-value < 0.05). Each model, one per cafeteria, was fitted using Rstudio software (R Core Team, 2021, Vienna, Austria). Additionally, we plotted the predicted probabilities using the ggplot2 package [55] from RStudio (http://www.r-project.org/index.html, accessed on 15 December 2021).

## 3. Results

### 3.1. Food Microbiological Analysis

The microbiological analyses of food samples are shown in Table 2 and Table S1. For operation A, 4/22 (18.2 %) of food samples exceeded the acceptable limits of APC (limits M), and 1/7 (14.3%) samples exceeded the acceptable limits for *Enterobacteriaceae*. In operation B, 8/36 (22.2%) food samples exceeded the APC limits, and none of the samples exceeded the legal limits for *Enterobacteriaceae.* Overall, 10/12 (83.3%) samples with APC levels exceeding the acceptable limits were RTE salads being served to customers at the sampling time.

We found *L. monocytogenes* in one food sample in each FSOp: In operation A, a food ingredient (frozen vegetable mix) was contaminated with the pathogen, while an RTE salad (cabbage and carrot mix) was contaminated in operation B. This salad sample also exceeded the limits for APC. We did not detect any other foodborne pathogens in any food sample analyzed (Table 2).

### 3.2. Evaluation of Contact Surfaces Contamination

All surface samples were analyzed for APC and the presence of *L. monocytogenes*. All four zones (1–4) were screened in both operations in the first sampling (visit 1). However, in visits 2 and 3, only surfaces from zones 1–3 were tested. We used the APC criteria by Losito et al. to define cleanliness in surfaces of zones 1 and 2 [46]. In operation A, 19 surface samples were obtained in zones 1 and 2. APC classified two of those samples as “improvable” (APC between 50–499 CFU/cm^2^), and five samples were classified as “not compliant” (APC counts > 500 CFU/cm^2^). In operation B, 35 samples were from zones 1 and 2; APC classified three of those as “improvable” and five as “not compliant” (Table 3). Three non-compliant surfaces were Gastronorm containers, used to hold foods, sampled after cleaning and sanitizing procedures (Table S3).

We identified *L. monocytogenes* only in Zone 3 samples in sampling visit 1; therefore, we decided to increase the number of samples from Zone 3 and eliminate Zone 4 samples in sampling visits 2 and 3 (Table 3 and Table S3).

*L. monocytogenes* was found on Zone 3 surfaces in both operations and all three visits. In FSOp-A, all *L. monocytogenes* contaminated surfaces were obtained from drains (Table S3). In visit 1, 2/13 Zone 3 samples contained the pathogen, and in visits 2 and 3, a single drain sample was contaminated with *L. monocytogenes*. In FSOp-B, *L. monocytogenes* was detected from drains, floors, and a worker’s boot cover samples. The pathogen was detected in 2/14 Zone 3 samples in visit 1 and 6/18 samples in both visits 2 and 3 (Table S3).

The logit model showed that the log APC value had a positive association with the probability of detecting *L. monocytogenes* on a surface (*p*-value < 0.05). The fit indicated that a one-unit increase in log APC would increase the probability of finding *L.* *monocytogenes* almost 2.5 times (Table 4; Figure S1).

### 3.3. L. monocytogenes Genome Characterization

We selected 18 *L. monocytogenes* isolates for whole-genome sequencing. These were obtained from food (n = 2) and the environment (n = 16). Sequenced isolates were obtained from both operations (A = 3; B = 15); isolates were obtained in visit 1 for operation A and all three operation B visits (Table 5). Genome sizes ranged from 2.99 Mb to 3.15 Mb, assembled genomes ranged from 25 to 182 contigs, and N50 varied from 153,922 to 596,977 (Table S4).

All isolates were *L. monocytogenes* lineage I, and they were of ST1/CC1, serogroup IVb (n = 1), ST2/CC2 of serogroup IVb (n = 10), and ST5/CC5 of serogroup IIb (n = 6). A single isolate was of the novel ST2349/CC5 of serogroup IIb. Bioinformatic prediction indicated that isolates from FSOp-A were all serogroup IVb, while FSOp-B had isolates of serogroup IVb or IIb.

We investigated the evolutionary history of the isolates to evaluate the persistence of strains within a facility. In a first phylogenetic reconstruction, including all 18 genomes, a total of 37,952 positions were found in the final SNP dataset. The phylogenetic reconstruction showed that genomes clustered based on serogroups: Cluster 1 included isolates of serogroup IVb, and Cluster 2 was composed of isolates of serogroup IIb (Supplementary Figure S2).

**Figure 2:**
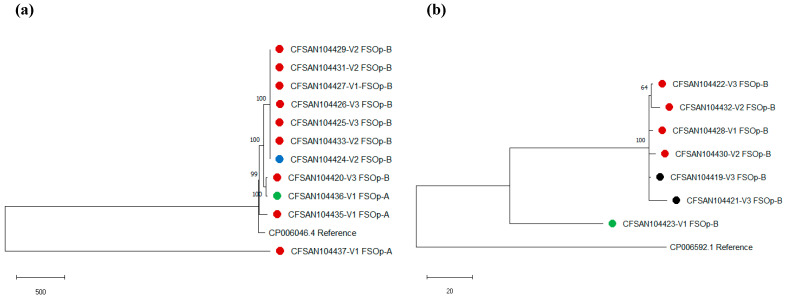
Neighbor-joining phylogeny of isolates based on SNP of *L. monocytogenes* by serogroup (**a**) IVb and (**b**) IIb. The circle color indicates isolation source: Red = drain; green = food; black = floor; and blue = boot cover.

An SNP analysis of isolates serogroup IVb from FSOp-A (n = 3) found over 100 SNP differences among genomes (Table S5, Figure 2a). The tree of strains serogroup IVb from operation B indicated that 7/8 isolates were closely related and included 1 to 7 SNPs differences (Table S5). Of those closely related isolates, 6/7 were obtained from drains in different FSOp-B areas (visits 1 through 3), and one was isolated from a boot cover (Figure 2). An *L. monocytogenes* serogroup IVb (FSOp-A) isolate obtained from an ingredient (frozen vegetable mix, CFSAN104436) was phylogenetically unrelated to isolates from the food facility (>170 SNPs). The phylogenetic analysis of serogroup IIb genomes indicated that 6/7 of those genomes were closely related; SNPs detected among those isolates ranged from 3 to 7 (Figure 2b; Table S5). By contrast, genome CFSAN104423, an *L. monocytogenes* serogroup IIb isolated from salad (novel ST2349/CC5), showed a range of 103 to 192 SNP differences with the remaining genomes of the same serogroup (Table S5). *L. monocytogenes* isolates obtained from food samples (CFSAN104436 and CFSAN104423) had over 100 SNP differences from the environmental isolates, indicating that it is unlikely that the environment was the source for the food contamination.

### 3.4. L. monocytogenes Phenotype Characterization

The analysis of QA-MIC revealed that 13/18 isolates were inhibited at 10 ppm, whereas five of the isolates were more susceptible to QA and showed a QA-MIC of 5 ppm (A17/612-3, A17/589-1, A17/606-3, A17/646 and A18/70-7) (Table 5). We searched for these elements -*qacH, qacC,* and *bcrABC-* in the *L. monocytogenes* genomes, and the *bcrA* gene was identified in all genomes. Interestingly, *bcrA* was the only gene present in 4 of 5 isolates with the lowest QA-MIC value (5 ppm). Isolates showing the highest QA-MIC values (10 ppm) encoded both *bcrB* and *bcrC* genes or the *qacH* gene (Table 5). The *qacC* gene was not found in any of the genomes analyzed.

**Figure 3:**
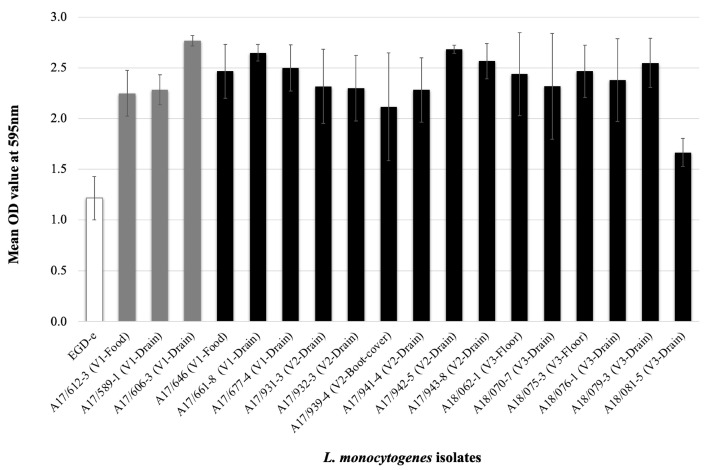
*L. monocytogenes* biofilm formation. Grey bars represent FSOp-A isolates, and black bars are FSOp-B isolates. *L. monocytogenes* EGD-e strain (serotype 1/2a) was used as a control.

All evaluated isolates (n = 18) formed biofilm at 37 °C. *L. monocytogenes* EGD-e, used as a control of biofilm formation, reached an optical density (OD_595nm_) value of 1.2. Our isolates showed higher biofilm production levels, ranging from OD_595nm_ 1.7 to 2.8 (Figure 3).

### 3.5. Audits: Evaluation of Good Manufacturing Practices

**Figure 4:**
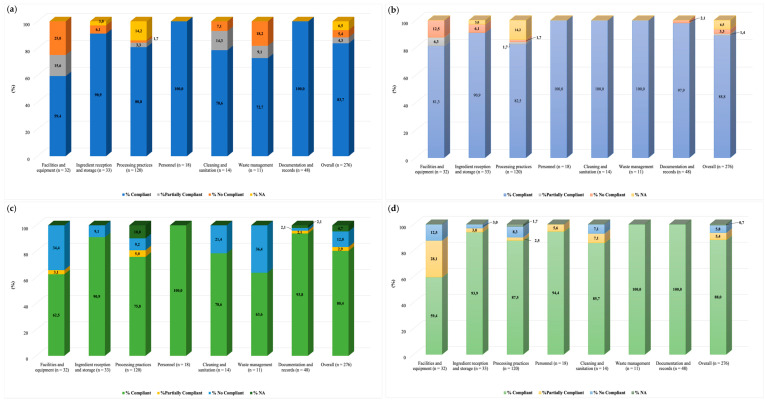
Audit results. Percent of compliant for each section analyzed. FSOp-A: (**a**) first audit (visit 1) and (**b**) second audit (visit 3). FSOp-B: (**c**) first audit (visit 1) and (**d**) second audit (visit 3).

We conducted two GMP audits (visit 1 and 3) in each FSOp. We observed improved overall conformity in both facilities between visits 1 and 3. FSOp-A improved compliance from 90% to 95% (“suitable”; 90-100% compliance to the checklist), and FSOp-B increased from 80% to 88% (“fair”; 76-89% compliance to the checklist) (Figure 4). The sections “facilities and equipment”, “cleaning and sanitization”, and “waste management” showed the lowest compliance (Figure 4).

“Facilities and equipment” was the section with the lowest overall compliance in the first audit; it was classified as “critical” (<65% compliance to the checklist) in both operations (FSOp-A = 59.4%; FSOp-B = 62.5%). Most deficiencies in both operations were related to subsections “location”, “roofing, lighting, windows, doors and stairs”, and “ventilation and air conditioning”. In both FSOp, surrounding areas, such as access roads, were unpaved and a potential source of contamination since dust was generated by vehicle and people movement. Ceiling conditions were also deficient; we observed water condensation in some areas and mold in FSOp-B. A detailed report of deficiencies and potential solutions was delivered to both management teams, and a second audit (during visit 3) was conducted in each facility. In this audit, FSOp-A scored 81.3% compliance (“fair”: 76-89% compliance) in this section, while operation B reduced compliance to 59.4% (“critical”; <65% compliance).

Overall, compliance with “cleaning and sanitization” was 78.6% for both FSOp-A and FSOp-B in the first audit, classified as “fair”, indicating that the possibility of food contamination exists, but it is controlled.

## 4. Discussion

Changes in lifestyles have determined that people eat away from home more frequently than ever before. Food services, such as cafeterias and restaurants, provide food to consumers and assure its wholesomeness. Therefore, they must comply with strict controls to ensure quality and safety. *L. monocytogenes* is a foodborne pathogen that adapts and survives to diverse environments in the food industry and can contaminate foods creating potential risks. Good manufacturing practices and environmental monitoring of surface contamination with the pathogen can help to mitigate the risks.

In this study, we evaluated food microbiology quality, food surfaces contamination, and GMP adherence for two FSOp in Santiago, Chile. We identified food samples with a higher APC level than allowed by the Chilean legislation. APC is an indicator of the hygienic quality of raw materials, storage problems, temperature abuse, and shelf life [56,57]. Notably, all ingredients used to make foods in both facilities were used before their best-by date; however, we cannot discard ingredient quality as a cause of high APC levels in these foods. As mentioned above, other failures such as temperature abuse might have contributed to these results. Moreover, two types of foods—frozen vegetables and an RTE salad—were contaminated with *L. monocytogenes*. Since listeriosis outbreaks have been linked to frozen corn and other frozen mixed vegetables, consumers and food handlers must be aware of the risks associated with improper handling of this type of food so that they can reduce the risk of infection and disease [58,59,60,61]. *L. monocytogenes* has also been isolated from RTE vegetable salads and fresh vegetables frequently used for salads, e.g., lettuce, parsley, spinach, carrot, and cabbage, among other vegetables [62,63,64,65]. Different foods have been linked to listeriosis outbreaks, including diverse fresh produce [66]. *L. monocytogenes* recently caused a foodborne outbreak linked to packaged leafy green salad affecting 33 people in the USA and Canada [67]. These results indicate that raw vegetables are a possible transmission vehicle for *L. monocytogenes,* and contamination in food processing plants must be addressed with strict programs, especially in plants processing RTE vegetables, as a means to ensure food safety [68].

High bacterial counts found on surfaces in direct contact with food have been linked to recontamination issues [69]. This investigation identified elevated aerobic bacterial counts in zones 1 and 2 in both FSOp. Although pathogenic bacteria were not found on these surfaces, effective disinfection protocols must be followed regularly to prevent pathogens from building biofilms.

*L. monocytogenes* is a psychrotrophic pathogen, and it can grow at temperatures as low as −1 °C [70,71,72]. These characteristics allow *L. monocytogenes* to proliferate in different ecosystems, including food processing facilities and diverse food matrices [18,73]. Therefore, low temperature is a futile control measure against this pathogen, and additional strategies are required to control it. Montero et al., (2015) reported that 29% of frozen vegetables and 2% of fresh vegetables in Chile were contaminated with *L. monocytogenes* [74]. The most significant outbreaks of listeriosis in Chile occurred between 2008–2009. In 2008, 165 cases and 14 deaths were reported associated with eating soft goat cheese, and in 2009 there were 73 cases and 17 deaths associated with sausage and other meat products [74]. After the 2008 goat cheese outbreak, *L. monocytogenes* was included in the Chilean RSA as a microorganism that must be controlled in foods [34]. Current Chilean regulation allows *L. monocytogenes* in foods that do not support bacterial growth, such as frozen vegetables, at levels up to 100 CFU/g. However, foods carrying *L. monocytogenes* and other foodborne pathogens might contribute to human disease by increasing the risk of cross-contamination of surfaces or other foods.

We also found that *L. monocytogenes* mainly was contaminating Zone 3 surfaces. Cleaning and sanitation practices are designed to remove food residues and reduce or eliminate microorganisms from food contact surfaces and the food processing environment. Therefore, the presence of *L. monocytogenes* in an FSOp may indicate unsatisfactory cleaning and sanitation procedures. Based on a survey of frozen food manufacturers, major areas of concern for finding *Listeria*-positive results in such facilities are floors, walls, and drains [75]. Drains might be considered growth niches or reservoirs where *L. monocytogenes* can survive sanitation. Although *L. monocytogenes* was not detected in Zones 1 and 2, its detection in drains may be used to predict the pathogen’s presence in other sites but does not indicate food systems failures [76]. Drains have also been classified as transfer points, i.e., sites with the potential to transfer microorganisms from one location to another [75]. Interestingly, isolates found in drains, floor, and a boot cover were genetically closely related, indicating that *L. monocytogenes* contamination was likely transferred via personnel walking in this operation. Most importantly, finding a highly related isolate in the boot cover might confirm that drains are acting as reservoirs and transfer zones for *L. monocytogenes*, increasing the risk for food contamination.

We observed that *L. monocytogenes* isolates obtained in these FSOp were largely clonal, and all belonged to lineage I. Worldwide, most listeriosis cases have been associated with genetic lineage I (serogroup IIb and IVb) [77]. Toledo et al., 2018, recently reported the genetic characterization of local isolates associated with two listeriosis outbreaks in Chile [53]. The analysis of these isolates indicated that 15 out of 22 (68 %) were serogroup IVb and from CC1. In this study, a single isolate belonging to CC1/ST1 was identified; it was obtained from a drain in FSOpA. Isolates belonging to CC1 have been isolated from different environments and described as hypervirulent and associated with outbreaks throughout the world [78,79].

In this study, *L. monocytogenes* serogroup IVb, CC2/ST2 was the isolates most frequently detected from drains (FSOpA and B). Similarly, the same molecular variant was predominant in an analysis performed in Poland in a study monitoring RTE foods, meat, and food environments [80]. It has also been reported that CC2 isolates were more salt-tolerant than isolates belonging to other CCs [81].

The lethal effect of quaternary ammonium compounds on bacteria is generated by disrupting the bacterial cell wall, resulting in cytosol loss. Antimicrobial activity may also involve disrupting and denaturing structural proteins and enzymes [82]. *L. monocytogenes* carry at least two genetic determinants that enhance QA tolerance, such as *qacH* and *bcrABC*, which codify for efflux pump proteins [83,84]. All the isolates CC5 (ST5) carrying the complete *bcrABC* operon showed the highest QA tolerance, similar to reports for an isolated obtained from a mushroom production factory in Netherland [85]. It has been reported that isolates carrying these genes can tolerate 5–13 ppm QA in vitro [86]. In 2008, Mullapudi et al. defined that isolates with an MIC of 10 ppm were resistant for QA [87]. In FSOpB we identified that 13/15 isolates showed an MIC of 10 ppm. Food processing plants use higher QA concentrations ranging from 200–1000 ppm QA compounds, acceptable ranges to control *L. monocytogenes* [86]. However, the incorrect use of QA due to insufficient rinsing may leave residues in sublethal concentrations at which *L. monocytogenes* strains harboring these genes may have a growth advantage [11,86,88]⁠.

In this study, audit non-conformities observed included dirty drains, which are prone to develop *L. monocytogenes* biofilms [76]. Biofilms can adhere to diverse surfaces commonly found in food-processing facilities such as stainless steel and diverse polymers [5,6,89]. Biofilms are an issue in food-processing environments due to their enhanced tolerance to disinfectants, providing adaptive resistance to environmental stresses such as osmotic stress, desiccation, and low-temperature [90,91]. Studies have shown that biofilm formation in processing plants tends to occur on drains and walls rather than food-contact surfaces [76,92,93]. Variables associated with biofilm production include the origin of the strain, serotype, temperature, and nutrients [94,95,96,97]. Six genes related to biofilm formation were retrieved from each *L. monocytogenes* genome. Studies revealed that mutant for these genes had shown reduced attachment and/or biofilm formation compared to wild-type strains [98,99,100,101,102]. However, we did not identify an association between the presence of these genes and biofilm production. These results suggest that the transcriptional response of biofilm-related genes varies between strains or other proteins involved in forming this structure [103,104].

In Europe, meals prepared away from home represent a risk factor for foodborne illnesses and have been implicated in up to 70% of foodborne outbreaks [105,106]. Likewise, in Chile, FSOp, such as cafeterias and restaurants, are responsible for 33% of reported foodborne outbreaks [107]. Therefore, adherence of these establishments to GMP significantly impacts public health [31,108,109]. The GMP checklist applied in this study evaluated seven different items. “Ingredient reception and storage”, “personnel”, and “documentation and records” were items with compliance greater than 90% in both FSOp. On the other side, “facilities and equipment”, “processing practices”, “cleaning and sanitation”, and “waste management” reached lower conformity percentages. Similar to our findings, facilities and waste management showed deficiencies in school cafeterias in Brazil during the evaluation of prerequisite program implementation. The authors mentioned that improving these nonconformities requires a high economic investment; however, the costs are justified because of benefits in food safety [110]. Studies estimate that the economic cost of restaurant-associated outbreaks can reach USD 2.6 million [111].

Waste management showed the best improvement during the second audit compared to the first visit. Initial compliance was 72.7% for FSOp-A and 63.6% for FSOp-B. Measures were suggested to increase compliance, such as scheduling regular waste removal programs and improving the waste storage area’s cleanliness and order. Both facilities achieved full compliance (100%) for waste management in the final audit. Proper cleaning and personal hygiene processes are critical in ensuring safe food production [112]. Failures in the latter have been associated with outbreaks, as occurred recently in a restaurant in Oman where 100 people were affected with a *Salmonella* infection [113]. Finally, foodservice staff plays an essential role in preventing food contamination. Even though the checklist determined that food handlers received sanitary food handling and hygiene training, personnel may not have understood why they performed certain activities. It is suggested that workers are educated on food safety regulations and management systems (HACCP). This knowledge, practice of food hygiene and safety, and personnel motivation may help to reduce the risk of foodborne disease transmission [105].

## 5. Conclusions

Genomic analysis of *L. monocytogenes* isolated from FSOp revealed that strains persist in Zone 3 areas for months, serving as pathogen reservoirs for the plant environment. The isolates tolerated up to 10 ppm of QA and formed biofilms, explaining their persistence in the environment despite cleaning and sanitation procedures. Since strains identified belonged to molecular serogroup IIb and IVb, which have been associated with human listeriosis, the persistence of these strains in FSOp might endanger consumers’ health. Therefore, GMP adherence and exhaustive cleaning protocols are required in FSOp to control the presence of *L. monocytogenes*. Multiple GMP failures were identified in these facilities, increasing the possibility for contamination of foods. GMP failures could provide the opportunity for contamination of food contact surfaces and food with this pathogen. Finally, we consider that the continuous training of food handlers improves the practices used and thus ensures food safety.

## Figures and Tables

**Table 1:** The number of surface samples collected for each zone in both FSOp.

FSOp	Zone	No Samples Analyzed			
		Visit 1	Visit 2	Visit 3	Total Samples
**A**	1	5	4	5	14
	2	1	2	2	5
	3	13	14	13	40
	4	1	0	0	1
	Total	20	20	20	60
**B**	1	6	6	6	18
	2	5	6	6	17
	3	14 *	18	18	50
	4	1	0	0	1
	Total	26 *	30	30	86

* On the first visit, four Zone 3 samples were mishandled during transportation to the laboratory, so they were discarded.

**Table 2:** Results of the microbiological analysis of food samples belonging to food services operations A and B. The parameters analyzed for each sample were defined according to Chilean Food Regulation (RSA). Grey squares indicate that the sample meets the level or the absence of the microorganism. Black squares indicate that the food sample exceeds the parameter level required by the RSA or indicate the microorganism’s presence (Food Standards Chile, 2019 [34]). White squares indicate parameters not tested, and the regulation does not require its testing. Numbers inside black or grey squares indicate the level found in the sample for each parameter.

	APC	*Enterobacteriaceae*	*E. coli*	*Salmonella* spp.	*S. aureus*	*C. perfringens*	*L. monocytogenes* *	RSA Requirements
**FSOp-A**								
**Sampling Visit 1**								
Frozen veg mix: Corn, carrot, and string beans	4.7 × 10^36^	10		ND			P	APC: M = 5 × 10^5^—Enterobacteriaceae: M = 5 × 10^4^—*Salmonella*: m = 0
**Sampling Visit 2**								
Salad: Potato salad	4.4 × 10^6^		<3	ND	<10		ND	APC: M = 10^6^—*E. coli*: M = 5 × 10^2^—*S. aureus*: M = 5 × 10^2^—*Salmonella*: m = 0
**Sampling Visit 3**								
Frozen broccoli	>10^6^	4.9 × 10^5^		ND			ND	APC: M = 5 × 10^5^—Enterobacteriaceae: M = 5 × 10^4^—*Salmonella*: m = 0
Frozen avocado purée	1.2 × 10^6^	10	<3	ND			ND	APC: M = 5 × 10^5^—Enterobacteriaceae: M = 5 × 10^5^ -*E. coli*: M 10^2^—*Salmonella*: m = 0
Salad: Celery	>10^6^		<3	ND	<10		ND	APC: M = 10^6^—*E. coli*: M = 5 × 10^2^—*S. aureus*: M = 5 × 10^2^—*Salmonella*: m = 0
**FSOp-B**								
**Sampling Visit 1**								
Salad: Cabbage and carrot mix	5 × 10^6^		<3	ND	<10		P	APC: M = 10^6^—*E. coli*: M = 5 × 10^2^—*S. aureus*: M = 5 × 10^2^—*Salmonella*: m = 0
**Sampling Visit 2**								
Salad: Cabbage and carrot mix	1.1 × 10^6^		<3	ND	<10		ND	APC: M = 10^6^—*E. coli*: M = 5 × 10^2^—*S. aureus*: M = 5 × 10^2^—*Salmonella*: m = 0
Salad: Boiled eggs and lettuce	1 × 10^6^		<3	ND	<10		ND	APC: M = 10^6^—*E. coli*: M = 5 × 10^2^—*S. aureus*: M = 5 × 10^2^—*Salmonella*: m = 0
Salad: Boiled string bean	2 × 10^6^		<3	ND	<10		ND	APC: M = 10^6^—*E. coli*: M = 5 × 10^2^—*S. aureus*: M = 5 × 10^2^—*Salmonella*: m = 0
Salad: Tomato and cilantro	5.4 × 10^5^		<3	ND	<10		ND	APC: M = 10^6^—*E. coli*: M = 5 × 10^2^—*S. aureus*: M = 5 × 10^2^—*Salmonella*: m = 0
Salad: Beef and mix vegetables	1.1 × 10^6^		<3	ND	<10	<10	ND	APC: M = 10^6^— *E. coli*: M = 5 × 10^2^—*S. aureus*: M = 5 × 10^2^—*Salmonella*: m = 0— *C. perfringens*: M = 5 × 10^2^.
Salad: Cucumber	2.2 × 10^7^		<3	ND	<10		ND	APC: M = 10^6^—*E. coli*: M = 5 × 10^2^—*S. aureus*: M = 5 × 10^2^—*Salmonella*: m = 0
**Sampling Visit 3**								
Dessert: Spanish custard (RTE)	5 × 10^6^		<3	ND	<10		ND	APC: M = 10^6^—*E. coli*: M = 5 × 10^2^—*S. aureus*: M = 5 × 10^2^—*Salmonella*: m = 0

* All samples were analyzed for *L. monocytogenes*. P = Presence. ND = Not detected.

**Table 3:** Number of non-compliant samples for APC counts and *L. monocytogenes* from environmental samples for FSOp-A and FSOp-B.

	FSOpA			FSOpB		
	Zone 1	Zone 2	Zone 3	Zone 1	Zone 2	Zone 3
Total samples	14	5	40	18	17	50
APC 50–499 CFU/cm^2^	1	1	NA	2	1	NA
APC >500 CFU/cm^2^	5	0	NA	3	2	NA
*L. monocytogenes*	ND	ND	4	ND	ND	14

NA: not applicable; ND: not detected.

**Table 4:** Generalized linear model for predicting *L. monocytogenes* on surfaces in FSOp-A and B.

FSOp	Predictor Variable	Estimate (Odds Ratio)	Pr (>[z]) ^&^
A	Log APC	1.499	0.168
B	Log APC	2.488	0.013 *

^&^ The *p*-value of the analysis. i.e., indicates the significance of the estimated values of the coefficients of the model. * *p*-value < 0.05.

**Table 5:** *L. monocytogenes* genomic characterization and MIC-QA values.

										QA Resistant Related Genes				Biofilm-Related Genes *		
FSOp	Visit	Strain	CFSAN Number	SRA	Source	Lineage	MLST	Serogroup	MIC-QA (ppm)	*bcrA*	*bcrB/bcrC*	*qacH*	*qacC*	*lmo0673*/*lmo2504*	*luxS*/*recO*	*lmo2026*/*lmo0435*
**A**	1	A17/612-3	CFSAN104436	SRR12957137	Frozen veg mi × 1	I	ST2/CC2	IVb	5	+				+	+	
	1	A17/589-1	CFSAN104437	SRR12957136	Drain	I	ST1/CC1	IVb	5	+				+	+	
	1	A17/606-3	CFSAN104435	SRR12957145	Drain	I	ST2/CC2	IVb	5	+				+	+	
**B**	1	A17/646	CFSAN104423	SRR12957144	RTE Salad^2^	I	ST2349 */CC5	IIb	5	+	+			+	+	
	1	A17/661-8	CFSAN104428	SRR12957143	Drain	I	ST5/CC5	IIb	10	+	+			+	+	
	1	A17/677-4	CFSAN104427	SRR12957142	Drain	I	ST2/CC2	IVb	10	+		+		+	+	
	2	A17/931-3	CFSAN104430	SRR12957141	Drain	I	ST5/CC5	IIb	10	+	+			+	+	
	2	A17/932-3	CFSAN104431	SRR12957140	Drain	I	ST2/CC2	IVb	10	+		+		+	+	
	2	A17/939-4	CFSAN104424	SRR12957139	Boot cover	I	ST2/CC2	IVb	10	+		+		+	+	
	2	A17/941-4	CFSAN104432	SRR12957138	Drain	I	ST5/CC5	IIb	10	+	+			+	+	
	2	A17/942-5	CFSAN104433	SRR12957135	Drain	I	ST2/CC2	IVb	10	+		+		+	+	
	2	A17/943-8	CFSAN104429	SRR12957134	Drain	I	ST2/CC2	IVb	10	+		+		+	+	
	3	A18/062-1	CFSAN104419	SRR12957151	Floor	I	ST5/CC5	IIb	10	+	+			+	+	
	3	A18/070-7	CFSAN104420	SRR12957150	Drain	I	ST2/CC2	IVb	5	+				+	+	
	3	A18/075-3	CFSAN104421	SRR12957149	Floor	I	ST5/CC5	IIb	10	+	+			+	+	
	3	A18/076-1	CFSAN104422	SRR12957148	Drain	I	ST5/CC5	IIb	10	+	+			+	+	
	3	A18/079-3	CFSAN104426	SRR12957147	Drain	I	ST2/CC2	IVb	10	+		+		+	+	
	3	A18/081-5	CFSAN104425	SRR12957146	Drain	I	ST2/CC2	IVb	10	+		+		+	+	

* *lmo0637*: flagellar protein gene; *lmo2504*: endopeptidase—nucleotide segregation gene; *luxS*: quorum sensing AI2 biosynthesis protein gene; *recO*: DNA gap repair protein gene; *lmo2026*: class I internalin (InlL) gene; *lmo0435*: biofilm-associated protein gene (BapL).

## Data Availability

Not applicable.

## Supplementary Materials

The following are available online at https://www.mdpi.com/article/10.3390/foods11060886/s1, Figure S1: Graphic representation of the predicted *L. monocytogenes* increases in FSOp-B surfaces according to Log APC. Figure S2: Neighbor-joining phylogeny based on SNP of *L. monocytogenes* (n = 18), isolated from FSOp-A (n = 3) and FSOp-B (n = 15). The circle color indicates isolation source—Red = drain; green = food; black = floor, and blue = boot cover title. Table S1: Results of microbiological analysis of foods samples. Table S2: Checklist applied in audits. Table S3: Results of microbiological surface analysis. Table S4: Genome data information. Table S5: SNP distance matrix for *L. monocytogenes* genomes of serogroups IVb and IIb.
