# Supplementary figures and images for: Evaluation of the Persistence and Characterization of Listeria monocytogenes in Foodservice Operations
Source: Foods. 2022 Mar 20;11(6):886. doi: 10.3390/foods11060886 (PMC8955912; doi:10.3390/foods11060886)

## Slide 1
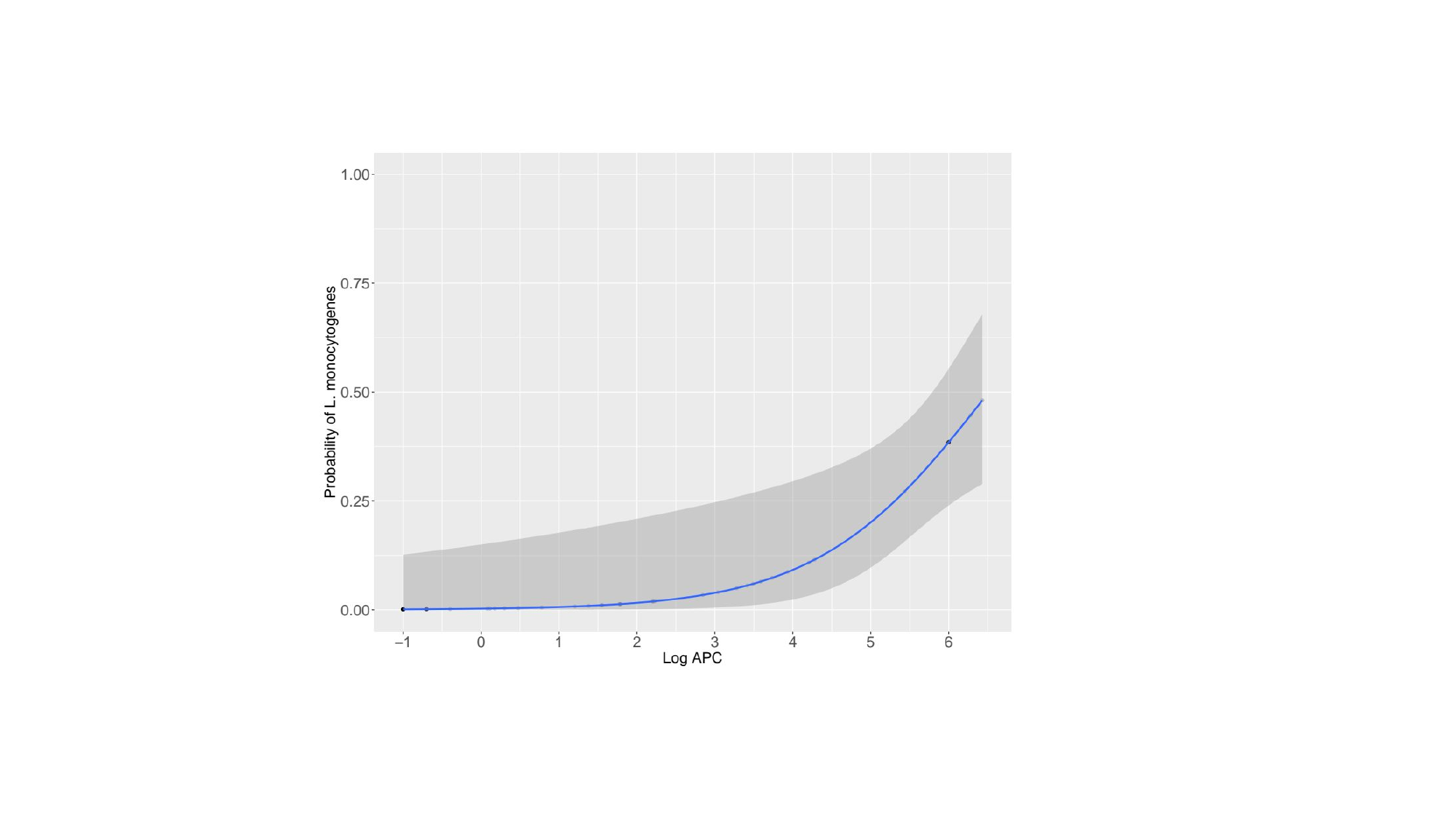

Supplement: Supplementary file 1 [file foods-11-00886-s001.zip › Suppl tables and fig/Suppl Fig 1_FOODS.pptx]
